# Supplementary figures and images for: Tracing RNA viruses associated with Nudibranchia gastropods
Source: PeerJ. 2022 May 13;10:e13410. doi: 10.7717/peerj.13410 (PMC9109684; doi:10.7717/peerj.13410)

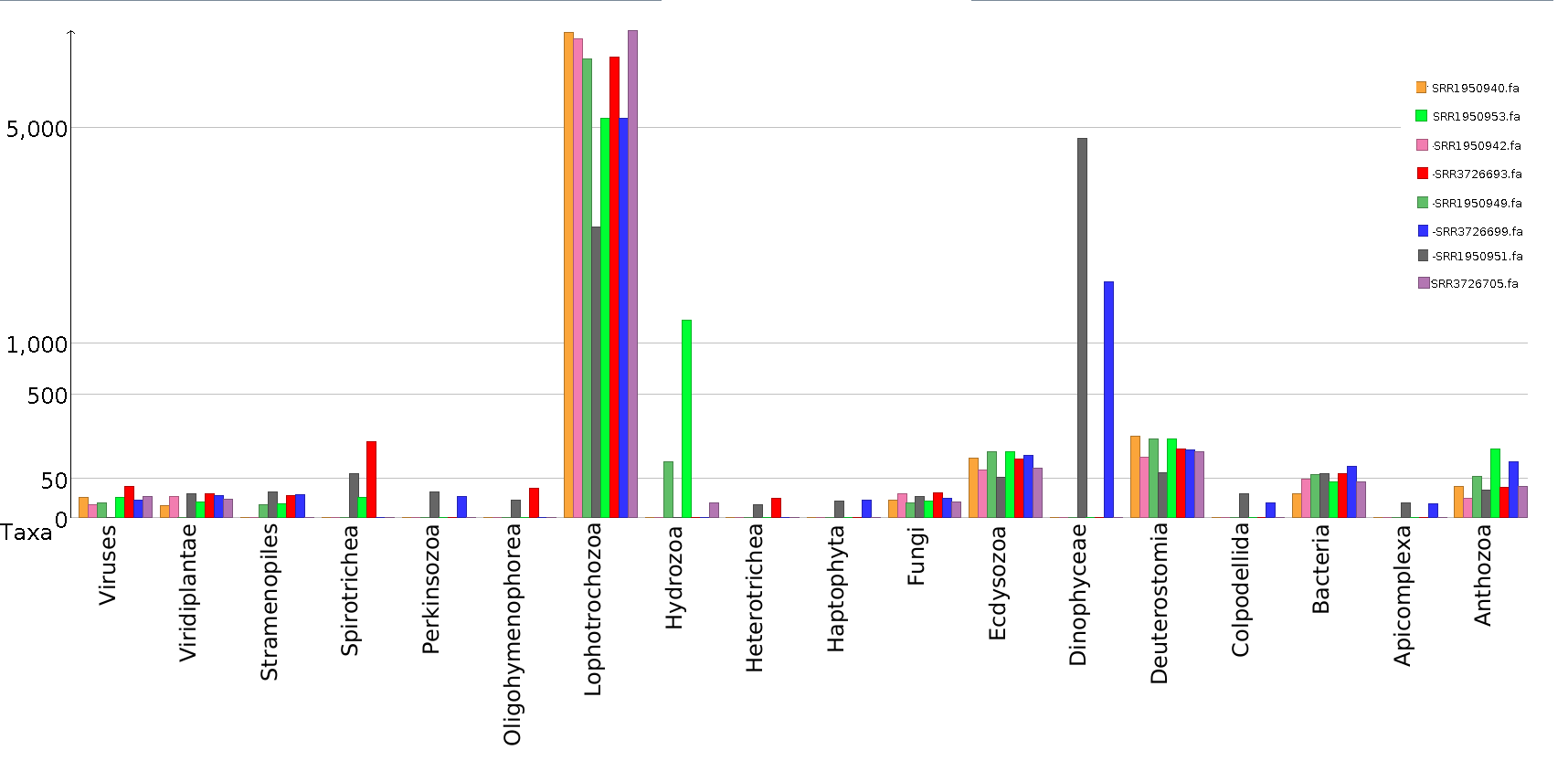

Supplement: Supplemental Information 3 [file peerj-10-13410-s003.png]
